# Worldwide threatening prevalence of carbapenem-resistant *Pseudomonas aeruginosa*

**DOI:** 10.1017/S0950268825100332

**Published:** 2025-09-19

**Authors:** Mohammad Hossein Ahmadi, Zeinab Fagheei Aghmiyuni, Shahriar Bakhti

**Affiliations:** Department of Microbiology, Faculty of Medicine, Shahed University, Tehran, Iran

**Keywords:** carbapenem resistance, doripenem, ertapenem, imipenem, meropenem, *P. aeruginosa*

## Abstract

β-Lactam/β-lactamase inhibitor combinations and carbapenems are the first-line treatments for multidrug-resistant *Pseudomonas aeruginosa* (*P. aeruginosa*) infections. However, carbapenem resistance is increasing globally at an alarming rate, which is especially concerning given the pivotal role of these agents. This study comprehensively evaluated the global distribution of carbapenem resistance in clinical *P. aeruginosa* isolates. The keywords including ‘*Pseudomonas’, P. aeruginosa’*, ‘*P. aeruginosa’*, ‘resistance’, ‘susceptibility’, ‘carbapenem antibiotics’, ‘carbapenems’, ‘imipenem’, ‘meropenem’, ‘ertapenem’, ‘doripenem’, as well as ‘prevalence’ and ‘incidence’ were searched in electronic databases as the appropriate keywords. After screening, 160 studies were excluded, with 87 eligible studies from diverse geographic regions retained for final analysis. A comprehensive meta-analysis was then conducted on the data collected. The mean resistance rates (95% CI) were 33.3% (imipenem), 23.3% (meropenem), 60.9% (ertapenem), and 36.7% (doripenem). The time trend analysis showed that the resistance to meropenem has increased from the year 1997 to 2023. Meta-analysis showed substantial heterogeneity (I^2^ = 92%, p < 0.05) but no significant publication bias by Egger’s or Begg’s test. Global carbapenem resistance is alarmingly high in clinical *P. aeruginosa* isolates. The increasing prevalence of carbapenem-resistant *P. aeruginosa* is a major global health threat requiring urgent action through new antimicrobials and improved antibiotic stewardship to protect these last-line drugs.

## Introduction

*Pseudomonas aeruginosa (P. aeruginosa)* is a Gram-negative, aerobic, non-fermentative bacillus and opportunistic pathogen [1–3]. This ubiquitous bacterium thrives in moist environments and poses a significant threat in healthcare settings [1, 2]. It commonly causes urinary tract infections, surgical site infections, healthcare-associated pneumonia, and bloodstream infections [1, 2, 4]. *P. aeruginosa* causes high mortality, especially in cystic fibrosis patients with chronic infections, burn victims, and the immunocompromised individuals [3–6] *P. aeruginosa* infections are managed through prevention, bacterial culture, and early antimicrobial therapy [6].

Antimicrobial therapy is the primary method for controlling bacterial infections [1, 3, 6]. Antipseudomonal antibiotics, including carbapenems, fluoroquinolones, and aminoglycosides, treat severe infections. Their misuse can increase antimicrobial resistance [1]. *P. aeruginosa* develops antibiotic resistance through drug inactivation, efflux pumps, and modifications to target sites or outer membranes [7].

*P. aeruginosa* strains with extensive drug resistance (XDR), defined as susceptibility to only one or two antibiotic classes, and multidrug resistance (MDR), resistance across ≥3 antimicrobial categories, pose an increasing global health threat [8]. The 2018 difficult-to-treat resistance classification for *P. aeruginosa* identifies isolates non-susceptible to all first-line antibiotics, including carbapenems (imipenem-cilastatin, meropenem), antipseudomonal β-lactams (ceftazidime, cefepime, and piperacillin-tazobactam), fluoroquinolones (ciprofloxacin, levofloxacin), and aztreonam [9]. A 2020 ECDC (European Center for Disease Prevention and Control) report showed concerning antibiotic resistance in *P. aeruginosa*, with 30.1% of isolates resistant to at least one agent in five core antimicrobial classes and 17.3% exhibiting cross-resistance to at least two [10].

Carbapenems are last-line antibiotics for severe multidrug-resistant infections [11]. Carbapenems are broad-spectrum ß-lactam antibiotics that include doripenem, ertapenem, imipenem, and meropenem [11]. *P. aeruginosa’s* carbapenemase genes confer carbapenem resistance, significantly reducing the effectiveness of common antipseudomonal agents [12]. Carbapenemases in *P. aeruginosa*, including Class A (KPC, GES), metallo-β-lactamases (IMP, NDM, SPM, VIM), and Class D (OXA-48) enzymes, exhibit regional variation [12]. Carbapenem-resistant *P. aeruginosa* (CRPA) infections exhibit resistance to multiple antibiotics [13]. In healthcare settings, CRPA spreads easily between patients *via* contaminated hands, surfaces, or equipment [6].

The World Health Organization (WHO) has nominated CRPA as a life-threatening-priority pathogen, highlighting the urgent need for new antimicrobial drugs [14]. Prevalence studies to prevent and monitor CRPA distribution exist, but published reports often focus on single healthcare facilities or geographical locations within countries [1]. Therefore, globally assessing the prevalence of CRPA strains in clinical isolates is crucial.

This study used a PRISMA-guided meta-analysis to estimate carbapenem resistance prevalence in clinical *P. aeruginosa* isolates worldwide [15].

## Methods

### Search strategy

This meta-analysis searched Web of Science, Scopus, PubMed, and Google Scholar from January 1997 to May 2023 for original English articles reporting the prevalence or incidence of carbapenem-resistant *P. aeruginosa.* Keywords included ‘*Pseudomonas*’, ‘*P. aeruginosa*’, ‘*P. aeruginosa*’, ‘resistance’, ‘susceptibility’, ‘carbapenem antibiotics’, ‘carbapenems’, ‘imipenem’, ‘meropenem’, ‘ertapenem’, ‘doripenem’, ‘prevalence’, and ‘incidence’, combined with Boolean operators (AND, OR).

### Study selection framework

This study included original research articles reporting *P. aeruginosa* resistance rates to carbapenems (imipenem, meropenem, ertapenem, and doripenem). Excluded were meta-analyses, reviews, theses, studies using non-human samples, articles lacking sufficient data for analysis, reports of genus-level (not species-level) antibiotic resistance, irrelevant titles, congress abstracts, and duplicate publications.

### Data extraction

The extracted dataset included publication year, study design, first author name, geographic region, participant demographics, specimen category, total isolates, resistance detection method, and resistant isolate counts per antibiotic. Two independent reviewers extracted data, resolving discrepancies through discussion.

### Statistical analysis

Carbapenem resistance prevalence was analysed using Comprehensive Meta-Analysis software (v3.7z, Bio stat) and expressed as pooled estimates with 95% confidence intervals. Heterogeneity was assessed using Cochran’s Q statistic and I^2^ statistic. If substantial heterogeneity was present (I^2^ > 50%), a random-effects model was used. Publication bias was evaluated *via* funnel plot symmetry, Begg’s test, and Egger’s test (p < 0.05). Each study was assigned a relative weight, and a meta-regression using the random-effect model (method of moments) has been performed [16, 17].

Bivariate linear regression using GraphPad Prism (v9.5) assessed temporal trends in *P. aeruginosa* carbapenem resistance (1997–2023), with average resistance rates and 95% confidence intervals reported. For antibiotics with sufficient data, the linear regression equation and R^2^ value were also calculated.

## Results

**Figure 1.**
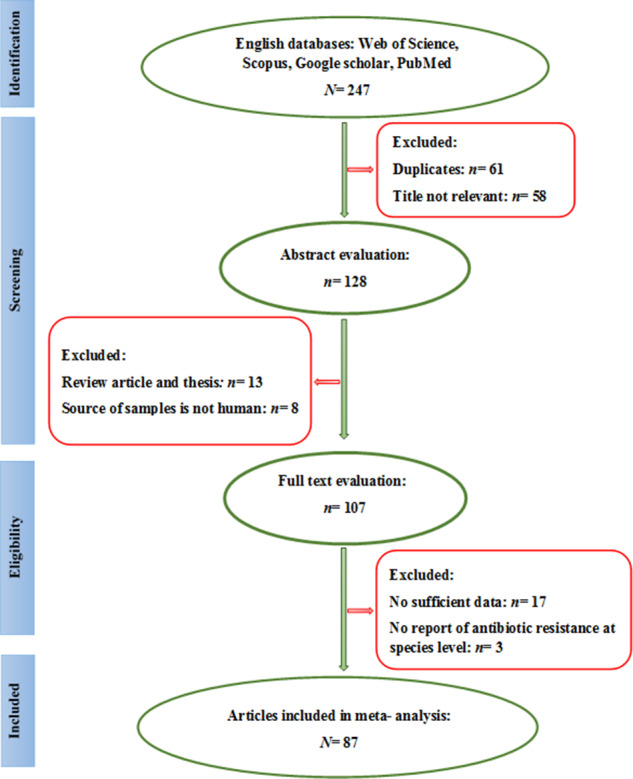
Flow chart of the literature search, systematic review and study selection.

Database searches (Web of Science, Scopus, Google Scholar, and PubMed) yielded 247 articles. After title screening, 119 articles were excluded (61 duplicates and 58 irrelevant titles). During subordinate screening, 21 publications were omitted due to either non-human specimen or article type (reviews). Full-text evaluation led to the exclusion of 20 more studies due to genus-level reporting (no species-level antibiotic resistance data) and/or deficient data. Ultimately, 87 articles published from January 1997 to May 2023 were included in the final analysis (Figure 1 and Table 1).

**Figure 2.**
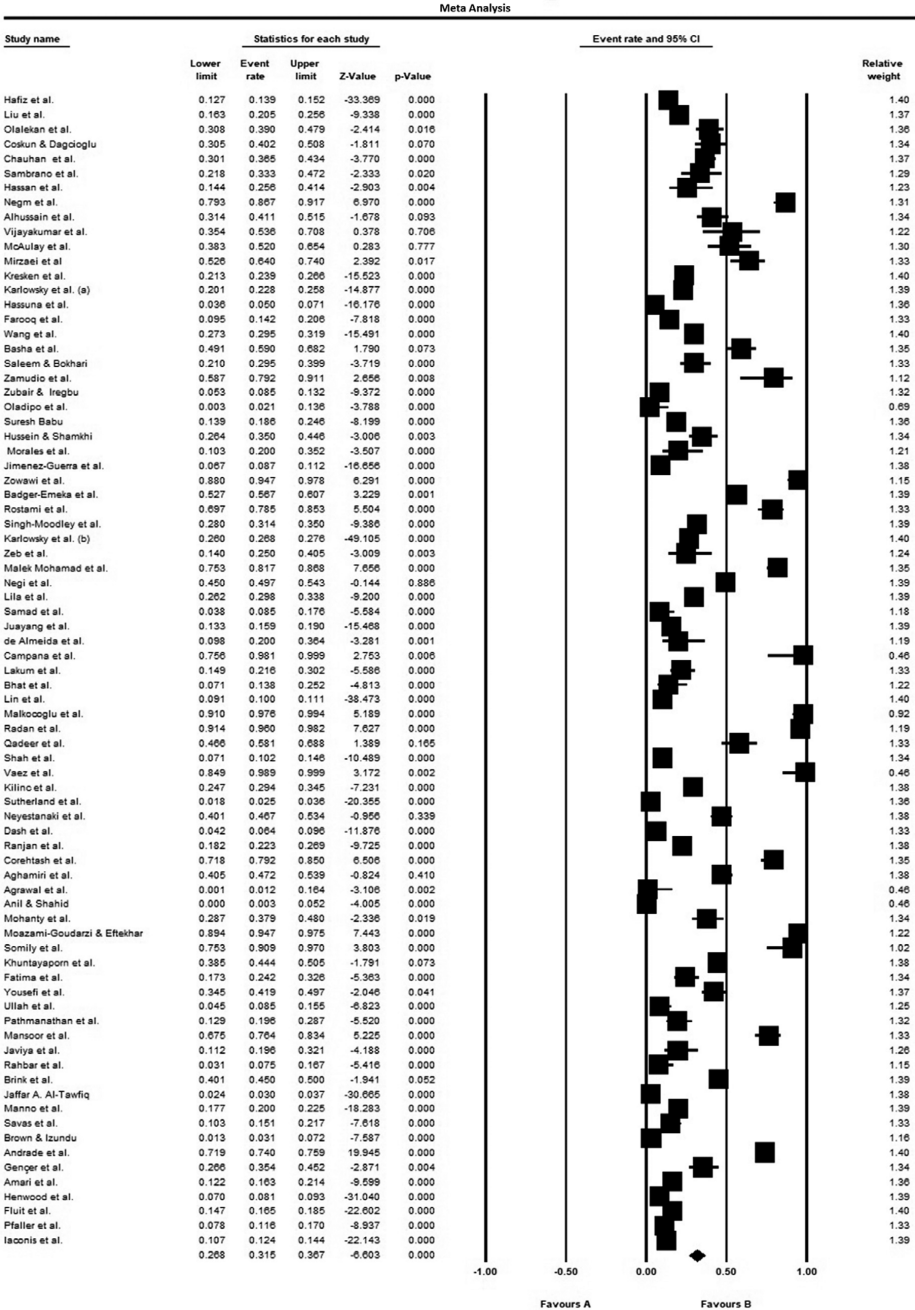
Forest plot of the meta-analysis of resistance rate of *P. aeruginosa* to imipenem.

**Figure 3.**
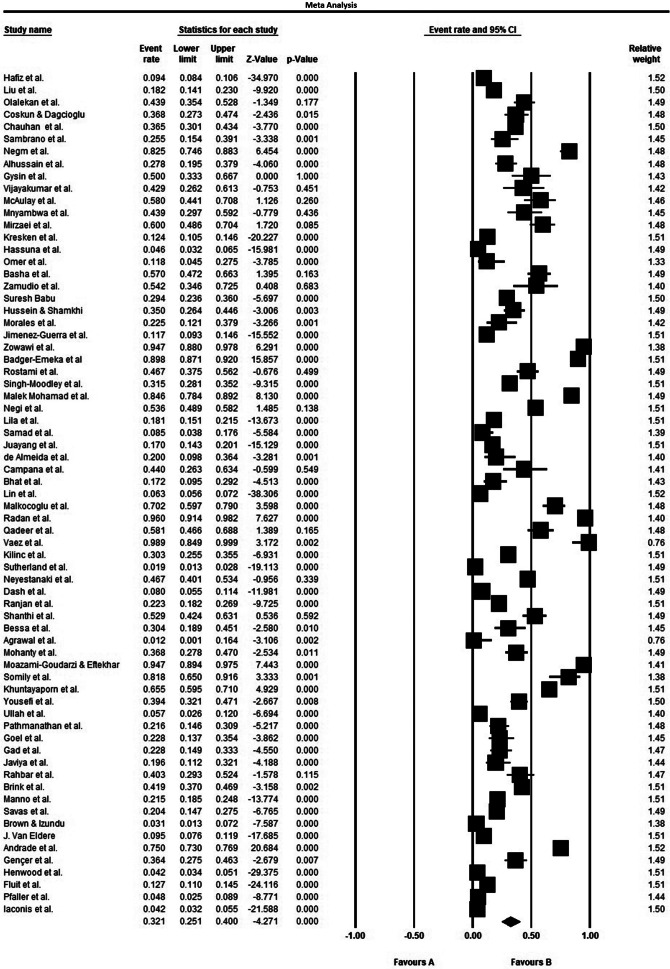
Forest plot of the meta-analysis of resistance rate of *P. aeruginosa* to meropenem.

**Figure 4.**
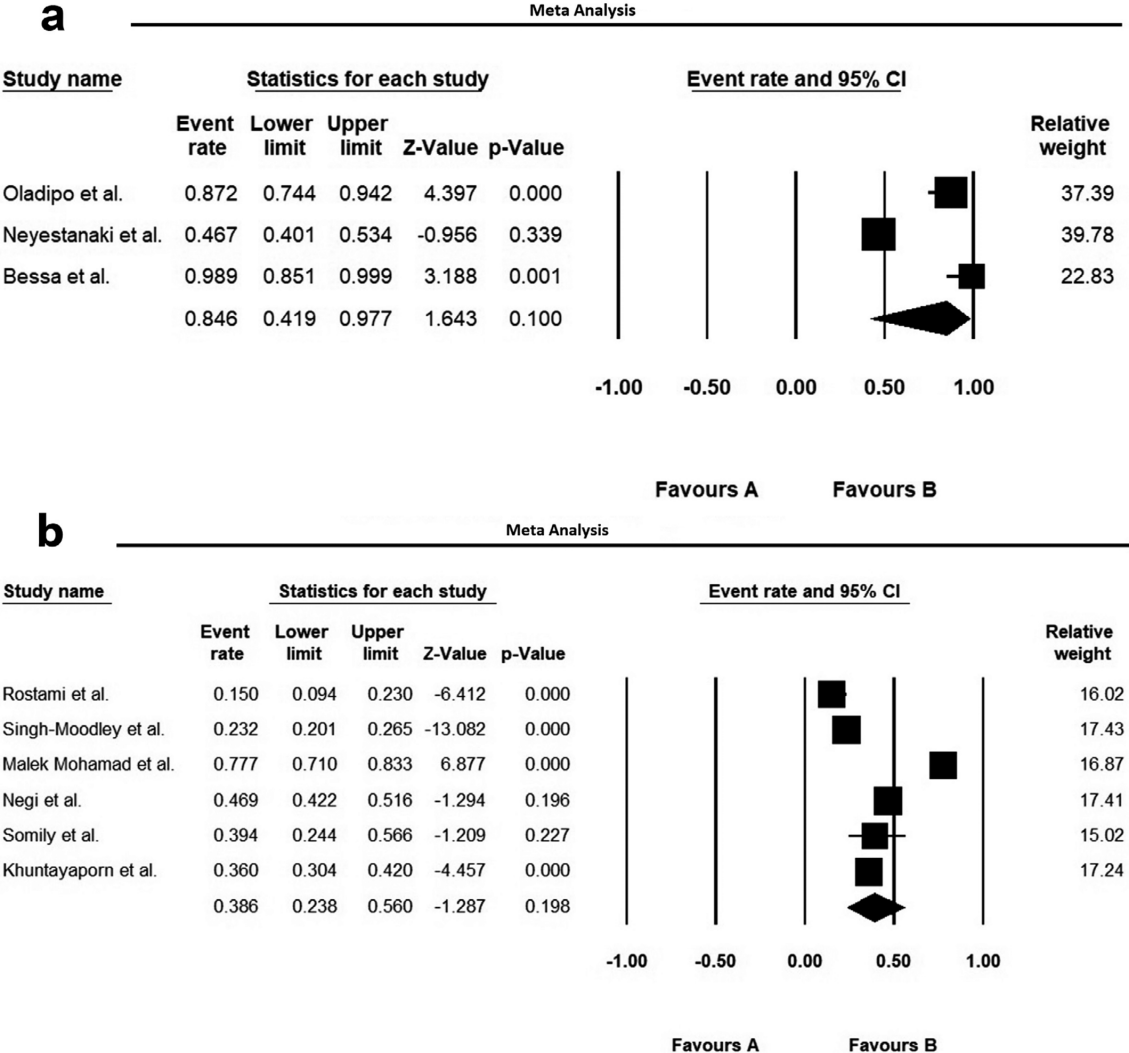
Forest plots of the meta-analysis of resistance rate to ertapenem (a) and doripenem (b) for *P. aeruginosa.*

The ultimate analysis merged data from 41 countries worldwide, including 49 Asian studies, 13 European investigations, 14 African reports, and 15 North/South American datasets (Table 1). The study population comprised diverse male and female patients from multiple care settings, outpatient clinics, general hospital wards, and intensive care units, all with confirmed *P. aeruginosa* infections. The included patients with hospital-acquired and healthcare-associated *P. aeruginosa* infections, across multiple clinical presentations: intra-abdominal, pulmonary, cutaneous/soft tissue, bloodstream, surgical site, urinary tract, as well as burn-related, cystic fibrosis-associated, COVID-19, and osteomyelitis cases (Table 1). Samples in the included studies consisted of blood, sputum, endotracheal secretions, bronchoalveolar lavage fluid, catheter tips, urine (mid-stream and catheterized), nasopharyngeal specimens, throat, ear, and eye swabs, abscess/wound samples, tissue, body fluid, and cerebrospinal fluid (CSF) (Table 1). Antimicrobial susceptibility testing in these studies focused on imipenem, meropenem, ertapenem, and doripenem. Mean resistance rates for *P. aeruginosa* isolates were 33.3% (imipenem), 23.3% (meropenem), 60.9% (ertapenem), and 36.7% (doripenem) (95% CI). Figures 2–4 show the forest plots of the meta-analysis for resistance rate of *P. aeruginosa* to the antibiotics. Table 2 shows the range and pooled resistance rates of *P. aeruginosa* isolates to antibiotics.

**Table 1.** Studies included in meta-analysis after final evaluation

Author (Reference)	Pub. year	Country	Source/specimen	No. of tested isolates	No. of resistant isolate (%)				Method of susceptibility testing	Disease/ complication/ participants
					Imipenem	Meropenem	Ertapenem	Doripenem		
Hafiz *et al.* [18]	2023	Saudi Arabia	Blood, sputum, ET, BAL, nasopharyngeal, throat swab, ear, and eye, urine (mid-stream urine and in-and-out catheters), abscess, wound, tissue, and body fluid, CSF	2,798	389 (13.9)	264 (9.4)	NM	NM	E-test	Patients infected by *P. aeruginosa*
Liu *et al.* [19]	2023	China	Sputum, alveolar lavage fluid, and other lower respiratory tract specimen (pleural fluid, flexible bronchoscopy with protected specimen brush, BAL, transbronchial biopsy, nonbronchoscopic BAL, or tracheobronchial aspirate in intubated patients)	292	60 (20.5)	53 (18.2)	NM	NM	Automated system and disk diffusion	Patients with nosocomial pneumonia
Olalekan *et al.* [20]	2023	Nigeria	Urine, wound swabs (including deep surgical swabs, diabetic foot swabs, burn wounds, crush injuries and ulcers), ear swabs, sputum/tracheotomy aspirates, and one vaginal swab	123	48 (39)	54 (44)	NM	NM	Vitek 2 automated system	Patients on admission and outpatients
Coşkun & Dagcioglu [21]	2023	Turkey	Respiratory samples, wound samples, urine, blood, and body fluid samples	87	35 (42.7)	32 (36.8)	NM	NM	Vitek 2 automated system	NS
Chauhan *et al.* [22]	2022	India	NS	200	73 (36)	73 (36)	NM	NM	Kirby–Bauer disk diffusion and E-Test	NS
Sambrano *et al.* [23]	2021	Panama	Blood, tracheal secretions, wound, eye and ear discharge, and ulcer)	51	17 (33)	13 (25)	NM	NM	Vitek 2 automated system	Patients admitted to Hospital
Hassan *et al.* [24]	2021	Egypt	Blood, urine, surgical site swabs, BAL/aspirate and others	39	10 (25.6)	NM	NM	NM	Kirby-Bauer disk diffusion	Patients with hospital acquired infections
Negm *et al.* [25]	2021	Egypt	Blood, urine, ET, sputum, BAL, central venous CT, pus, wounds/surgical site swabs, CSF, and peritoneal and pleural fluids.	120	104 (87.2)	99 (82.7)	NM	NM	Vitek 2 automated system	ICU patients
Alhussain *et al.* [26]	2021	Saudi Arabia	Blood, urine, wound, sputum	90	37 (41.1)	25 (27.8)	NM	NM	Vitek 2 automated system	Hospital acquired infections
Gysin *et al.* [27]	2021	Switzerland	Respiratory samples	32	NM	16 (50)	NM	NM	Kirby-Bauer disk diffusion	COVID–19 patients
Vijayakumar *et al.* [28]	2021	India	Pus samples from deep tissue and bone tissue	28	15 (53.6)	12 (42.9)	NM	NM	Kirby Bauer disc diffusion	Osteomyelitis patients
McAulay *et al.* [29]	2021	Haiti	Wound swabs, blood culture, joint, pus	50	26 (52)	29 (58)	NM	NM	Automated MicroScan WalkAway plus System (Beckman Coulter)	Patients at five healthcare facilities
Mnyambwa *et al.* [30]	2021	Tanzania	Blood, ear pus, wound pus, urine	41	NM	18 (66.7)	NM	NM	Kirby-Bauer disc diffusion	Outpatients and inpatients as part of each hospital’s routine clinical care
Mirzaei *et al.* [31]	2020	Iran	Wound, urine, sputum, blood, faeces, and trachea	75	48 (64)	45 (60)	NM	NM	Kirby-Bauer disk diffusion	NS
Kresken *et al.* [32]	2020	Germany	blood, respiratory tract, intra-abdominal specimen, and urine	985	235 (23.9)	122 (12.4)	NM	NM	Broth microdilution	Hospitalized patients
Karlowsky *et al. (a)* [33]	2020	United States	Intra-abdominal and urinary tract samples	846	193 (22.8)	NM	NM	NM	Broth microdilution	Intra-abdominal & UTIs
Hassuna *et al* [34]	2020	Egypt	Urine, sputum, wound, tracheal aspirates, bloodstream infections, and BAL samples	634	32 (5)	29 (4.5)	NM	NM	Agar dilution	NS
Omer *et al.* [35]	2020	Sudan	Wound	34	NM	4 (11.8)	NM	NM	Kirby–Bauer disc diffusion	Patients with diabetic septic foot wounds
Farooq *et al.* [36]	2020	Pakistan	Sputum	155	22 (14)	NM	NM	NM	Kirby-Bauer disk diffusion	Patients of Respiratory Tract Infections
Wang *et al.* [37]	2020	China	Sputum, urine, blood, pus, and other clinical specimens	1,524	450 (29.5)	NM	NM	NM	Vitek 2 automated system	Patients from Seven acute care hospitals
Basha *et al.* [38]	2020	Egypt	NS	100	59 (59)	57 (57)	NM	NM	Kirby-Bauer disk diffusion	NS
Saleem & Bokhari [39]	2020	Pakistan	Pus, wound, blood, urine	88	26 (30.2)	NM	NM	NM	Kirby-Bauer disk diffusion	Hospitalized patients and patients with pus wound, bacteraemia, & ear infection
Zamudio *et al.* [40]	2019	United Kingdom	Sputum	24	19 (79.2)	13 (54.2)	NM	NM	E-test	Cystic fibrosis
Zubair & Iregbu [41]	2018	Nigeria	Wound swab/ biopsy, ear swab, urine, blood, eye swab, sputum, nasal swab, and bone tissue	200	17 (8.5)	NM	NM	NM	Kirby–Bauer disc diffusion	All samples except stool
Oladipo *et al.* [42]	2018	Nigeria	NS	47	1 (2.1)	NM	41 (87.2)	NM	Kirby–Bauer disc diffusion	NS
Suresh Babu [43]	2018	India	Pus and discharges from various wound infections	204	38 (18.7)	60 (29.5)	NM	NM	Kirby- Bauer disc diffusion	Patients with wound infections
Hussein & Shamkhi [44]	2018	Iraq	Burn swab, sputum, ear swabs	103	36 (34.95)	36 (34.9)	NM	NM	Kirby-Bauer disk diffusion	Patients admitted to hospital
Morales *et al.* [45]	2018	Colombia	NS	40	8 (20)	9 (22.5)	NM	NM	Vitek 2 automated system	Infected patients
Jimenez-Guerra *et al.* [46]	2018	Spain	Urine or samples from indwelling or intermittent catheters	632 573	55 (8.7) NM	NM 67 (11.7)	NM	NM	E-test	Patients with UTI
Zowawi *et al.* [47]	2018	Countries of the Persian Gulf Cooperation Council	NS	95	90 (94.7)	90 (94.7)	NM	NM	Kirby-Bauer disk diffusion	NS
Badger-Emeka *et al.* [48]	2018	Saudi Arabia	Blood, urine, endotracheal aspirates, sputum, and wound	580	329 (56.7)	521 (89.9)	NM	NM	Kirby-Bauer disk diffusion	Patients who were in ICUs
Rostami *et al.* [49]	2018	Iran	Burn wound, blood, urine	107	84 (78.5)	50 (46.7)	NM	16 (15)	Kirby-Bauer disk diffusion & E-test	Burn patients
Singh-Moodley *et al.* [50]	2018	Africa	Blood	669	210 (31)	211 (32)	NM	155 (23)	MicroScan Walkaway system	Patients with bacteraemia
Karlowsky *et al.* (b) [51]	2018	Several countries from seven global regions	Intra-abdominal, urinary tract, and lower respiratory tract	12,170	3,261 (26.8)	NM	NM	NM	Broth microdilution	NS
Zeb *et al.* [52]	2017	Pakistan	Blood, urine, and tracheal secretion	40	10 (25)	NM	NM	NM	Kirby-Bauer disk diffusion	Patients who were hospitalized for more than week duration
Malek Mohamad *et al.* [53]	2017	Iran	Wound, blood, urine, respiratory specimens, burn wound, CSF, and others	175	143 (81.7)	148 (84.6)	NM	136 (77.7)	Kirby-Bauer disk diffusion	NS
Negi *et al.* [54]	2017	India	Sputum, endotracheal aspirate, BAL, & mini BAL	435	216 (49.6)	233 (53.5)	NM	204 (46.8)	E-test	Patients with suspected respiratory tract infections
Lila *et al.* [55]	2017	Kosovo	Tracheostomy tubes, endotracheal aspirate, wounds, blood, punctate of pleural liquid, thoracic & abdominal drain swab, CSF, and CT	553	165 (29.8)	100 (18)	NM	NM	Kirby-Bauer disk diffusion	Hospitalized patients
Samad *et al.* [56]	2017	Pakistan	Sputum samples	71	6 (8.5)	6 (8.4)	NM	NM	Kirby-Bauer disk diffusion	Patients with respiratory tract infections
Juayang *et al.* [57]	2017	Philippines	Blood, urine, wounds, abscesses, transudates and exudates, sputum, and tracheal aspirates	646	103 (16)	110 (17.1)	NM	NM	Kirby-Bauer disk diffusion	Patients with infections caused by *P. aeruginosa*
de Almeida *et al.* [58]	2017	Brazil	Burn wounds	35	7 (20)	7 (20)	NM	NM	Kirby-Bauer disk diffusion	Burn patients
Campana *et al.* [59]	2017	Brazil	NS	25	25 (100)	11 (44)	NM	NM	Broth microdilution	Hospitalized patients
Lakum *et al.* [60]	2016	India	Pus/wound, sputum, urine, CT, body fluids, and ET	111	24 (21.6)	NM	NM	NM	Kirby-Bauer disk diffusion	Patients at the tertiary care centre, Hospital
Bhat *et al. [61]*	2016	India	Blood, urine, skin/soft tissue, and respiratory samples	58	8 (13.8)	10 (17.2)	NM	NM	Disc diffusion	Patients from medical and surgical oncology units
Lin *et al.* [62]	2016	Taiwan	NS	3,408	342 (10)	216 (6.3)	NM	NM	Broth microdilution	Hospitalized patients
Malkocoglu *et al.* [63]	2016	Turkey	Respiratory specimens, urine, wound and soft tissue specimens, blood, sterile body fluid, catheters, and CSF	84	82 (97.6)	59 (70.2)	NM	NM	BD Phoenix Automatized AST system or Kirby-Bauer disk diffusion	NS
Radan *et al.* [64]	2016	Iran	Burn wound	150	144 (96)	144 (96)	NM	NM	Kirby-Bauer disk diffusion	Burn patients
Qadeer *et al.* [65]	2016	Pakistan	Blood, urine, ET, CT, tissue, pus swabs, CSF, ascites, BAL, and pleural fluid	74	43 (59)	43 (59)	NM	NM	Kirby-Bauer disk diffusion	ICU patients
Shah *et al.* [66]	2015	Pakistan	Urine	254	26 (10.4)	NM	NM	NM	Kirby-Bauer’s disk diffusion	Patients with UTI
Vaez *et al.* [67]	2015	Iran	Wound swabs	45	45 (100)	45 (100)	NM	NM	Kirby-Bauer’s disk diffusion	Burned patients admitted to ICU
Kılınç *et al.* [68]	2015	Turkey	Urinary system, respiratory system, surgical wounds, and blood samples	327	96 (30)	99 (31.2)	NM	NM	Vitek 2 automated system	Patients in ICU, chest service, pediatrics, otorhinolaryngology, surgery & urology wards
Sutherland *et al.* [69]	2015	United States	Blood, respiratory tract, wound, body fluid, and others	1,257	32 (2.5)	24 (1.9)	NM	NM	Broth microdilution	Adult hospitalized patients
Neyestanaki *et al.* [70]	2014	Iran	Burn wounds samples	214	100 (46.7)	100 (46.7)	100 (46.7)	NM	Kirby-Bauer disk diffusion	Burn patients
Dash *et al.* [71]	2014	India	Pus/swab, urine, sputum, blood, CSF, pleural fluid, peritoneal fluid, tissue biopsies, and BAL	327	21 (6.4)	26 (8)	NM	NM	Kirby-Bauer disc diffusion	Patients admitted in the hospital and out-patients
Ranjan *et al.* [72]	2014	India	Pus swab, endotracheal lavages, blood, sputum, CT, and urine	350	78 (22.2)	78 (22.2)	NM	NM	Kirby Bauer disk diffusion	All medical and surgical departments
Corehtash *et al.* [73]	2014	Iran	Burn wound infections	144	114 (79.2)	NM	NM	NM	Kirby Bauer disk diffusion	Burn patients
Aghamiri *et al.* [74]	2014	Iran	Wound, blood, urine, trachea, sputum, pleural fluid, eye, catheter, and larynges samples	212	100 (47.16)	NM	NM	NM	Agar dilution method	Patients admitted to hospitals
Shanthi *et al.* [75]	2013	India	Sputum, ET, pus, and blood swabs, BAL, and urine	87	NM	46 (52.9)	NM	NM	Kirby-Bauer disk diffusion	Burn wound infections & ICU patients
Bessa *et al.* [76]	2013	Italy	Wound	46	NM	14 (30·4)	46 (100)	NM	Vitek 2 automated system	Patients with infected wounds
Agrawal *et al.* [77]	2013	India	Ear discharge	41	0 (0)	0 (0)	NM	NM	Kirby-Bauer disk diffusion	Patients with otitis media and ear discharge
Anil & Shahid [78]	2013	Nepal	Pus/wound, sputum, urine, tracheal aspirates, central venous, CT, BAL, catheters, and high vaginal swabs	145	0 (0)	NM	NM	NM	Kirby-Bauer disk diffusion	Hospitalized patients
Mohanty *et al.* [79]	2013	India	Blood	95	36 (37.9)	35 (36.8)	NM	NM	Kirby-Bauer disk diffusion	Patients admitted to various wards of the hospital
Moazami-Goudarzi & Eftekhar [80]	2013	Iran	Wounds, blood, subclavian catheters, urine	133	126 (94.7)	126 (94.7)	NM	NM	Kirby-Bauer disk diffusion	Burn patients
Somily *et al.* [81]	2012	Saudi Arabia.	Wounds, respiratory samples (sputum, BAL and tracheal aspirates), urine, superficial swabs, and CT	33	30 (90.9)	27 (81.8)	NM	13 (39.4)	E-test	Patients at King Khalid University Hospital
Khuntayaporn *et al.* [82]	2012	Thailand	NS	261	116 (44.4)	171 (65.5)	NM	94 (36)	Kirby-Bauer disk diffusion	NS
Fatima *et al.* [83]	2012	Pakistan	Sputum samples	120	29 (24)	NM	NM	NM	Kirby-Bauer disk diffusion	Adult patients attended/admitted in pulmonary tract infections department
Yousefi *et al.* [84]	2010	Iran	Bronchial fluid, blood culture, catheter, CSF, ear, pleural fluid, sputum, urine, and wound	160	67 (41.8)	63 (39.3)	NM	NM	Kirby-Bauer disk diffusion	Hospitalized patients and out-patients
Ullah *et al.* [85]	2009	Pakistan	Wound swabs	106	9 (8.5)	6 (5.7)	NM	NM	Kirby-Bauer disk diffusion	Burn patients
Pathmanathan *et al.* [86]	2009	Malaysia	Respiratory tract, wound swab, urine, blood, tissue, and CSF	97	19 (19.5)	21 (21.6)	NM	NM	E-test	Hospitalized patients
Mansoor *et al.* [87]	2009	Pakistan	Discharging ear	110	84 (76)	NM	NM	NM	Agar dilution method	Patients with unilateral or bilateral chronic suppurative otitis media
Goel *et al.* [88]	2009	India	Trans tracheal or bronchial aspirates	57	NM	13 (22.8)	NM	NM	Kirby-Bauer disk diffusion	ventilated patients in ICU
Gad *et al.* [89]	2008	Egypt	Urine, wound, abscess and burn exudates sputum and purulent ear discharge	79	NM	18 (22.7)	NM	NM	Agar dilution method	Patients with UTI, patients with skin infection, patients with respiratory tract infection
Javiya *et al.* [90]	2008	India	Sputum, ET, BAL, blood, urine, body tissues, pus, semen, CSF, body fluids (peritoneal fluid), and costal bronchial secretions	56	11 (19.6)	11 (19.6)	NM	NM	Kirby–Bauer disc diffusion	Patients with *P. aeruginosa* infections
Rahbar *et al.* [91]	2008	Iran	Tracheal tube aspirates, urine, wound, blood, and other sterile body fluids	67	5 (7.5)	27 (40.2)	NM	NM	Kirby-Bauer disk diffusion and E-test	Hospitalized patients
Brink *et al.* [92]	2007	Africa	Blood	382	172 (45)	160 (42)	NM	NM	Kirby-Bauer disk diffusion	Hospitalized patients
Jaffar A. Al-Tawfiq [93]	2007	Saudi Arabia.	Respiratory, wound, urine, blood	2,679	80 (3)	NM	NM	NM	Vitek automated system	Inpatients and outpatients
Manno *et al.* [94]	2005	Italy	Sputa or oropharyngeal swabs	1,086 670	217 (20) NM	NM 144 (21.5)	NM NM	NM NM	Kirby-Bauer disk diffusion	Cystic fibrosis patients
Savas *et al.* [95]	2005	Turkey	Urinary, and surgical site infections, burns, tracheal aspiration samples	152	23 (15)	31 (20.4)	NM	NM	Kirby-Bauer disc diffusion	ICU and burns unit patients
Brown & Izundu [96]	2004	Jamaica	Wound swabs, high vaginal swabs, ear swabs	162	5 (3)	5 (3)	NM	NM	Kirby-Bauer disk diffusion	Hospitalized and outpatients
J. Van Eldere [97]	2003	Belgium	Respiratory samples, urinary samples, blood, and other sources	716	NM	68 (9.5)	NM	NM	NM	hospitalized Patients
Andrade *et al.* [98]	2003	six countries: Argentina, Brazil, Chile, Colombia, Mexico and Uruguay	Respiratory tract, bloodstream, skin and soft tissue, and urinary tract	1894	1,401 (74)	1,420 (74.9)	NM	NM	Broth microdilution	Bloodstream infections, pneumonia, skin and soft tissue infections, and UTIs
Gençer *et al.* [99]	2002	Turkey	Surgical wound or abscess, urine, blood, trans tracheal aspirates, pleural fluid, CSF fluid, ear, and peritoneal fluid.	99	35 (35.3)	36 (36.3)	NM	NM	E-test	Hospitalized patients
Amari *et al.* [100]	2001	Switzerland	Blood	252	41 (16.2)	NM	NM	NM	Disk diffusion	Patients with bacteraemia
Henwood *et al.* [101]	2001	UK	Respiratory tract, urinary tract, ear eye, device tips, and lines, wounds, blood, others	2,194	178 (8.1)	92 (4.2)	NM	NM	BSAC disc testing method	Patients with clinically significant infections
Fluit *et al.* [102]	2000	The 25 participating European hospital	Blood, respiratory tract, wound, urinary tract samples	1,411	233 (16.5)	179 (12.7)	NM	NM	Broth microdilution	North American SENTRY participants
Pfaller *et al.* [103]	1998	United States and Canada	Blood	189	22 (12)	9 (4.8)	NM	NM	Broth microdilution	Patients with bloodstream infections
Iaconis *et al.* [104]	1997	United States	Bone and /or skin structure, lower respiratory tract, urinary tract, or intra-abdominal infections samples	1,182	147 (12.5)	50 (4.2)	NM	NM	Agar dilution method and broth microdilution	Hospitalized patients

Abbreviations: NM: not measured; NS: not specified; ICU: Intensive care unit; CSF: cerebrospinal fluid; ET: Endo-tracheal tract secretions, BAL: bronchoalveolar lavage; CT: Catheter tip; UTI: urinary tract infection.

**Table 2.** Meta-analysis results for resistance rate of *Pseudomonas aeruginosa* in included studies

Clinical isolate	Number of studies	Antibiotic	Resistance rate (%) (95% CI)			Heterogeneity test		Begg’s test^b^ *p*-value (two-tailed)		Egger’s test^c^ *p*-value (two-tailed)
			Min	Max	Pooled^a^ (range)	*I* ^2^ (%)	*p*-value	a	b	
*P. 0aeruginosa*	79	Imipenem	0	100	0.3 (0.2–0.4)	98.6	<0.001	0.2	0.2	0.5
	69	Meropenem	0	100	0.3 (0.2–0.4)	98.9	<0.001	0.2	0.3	0.4
	3	Ertapenem	46.7	100	0.8 (0.4–1.0)	93.3	<0.001	0.6	1.0	0.2
	6	Doripenem	15	77.7	0.4 (0.2–0.6)	97.3	<0.001	0.6	0.7	0.8

*Note:* a: Kendall’s tau without continuity correction; b: Kendall’s tau with continuity correction.

a Pooled resistance rate (based on random effects).

b Begg and Mazumdar rank correlation.

c Egger’s regression intercept.

**Figure 5.**
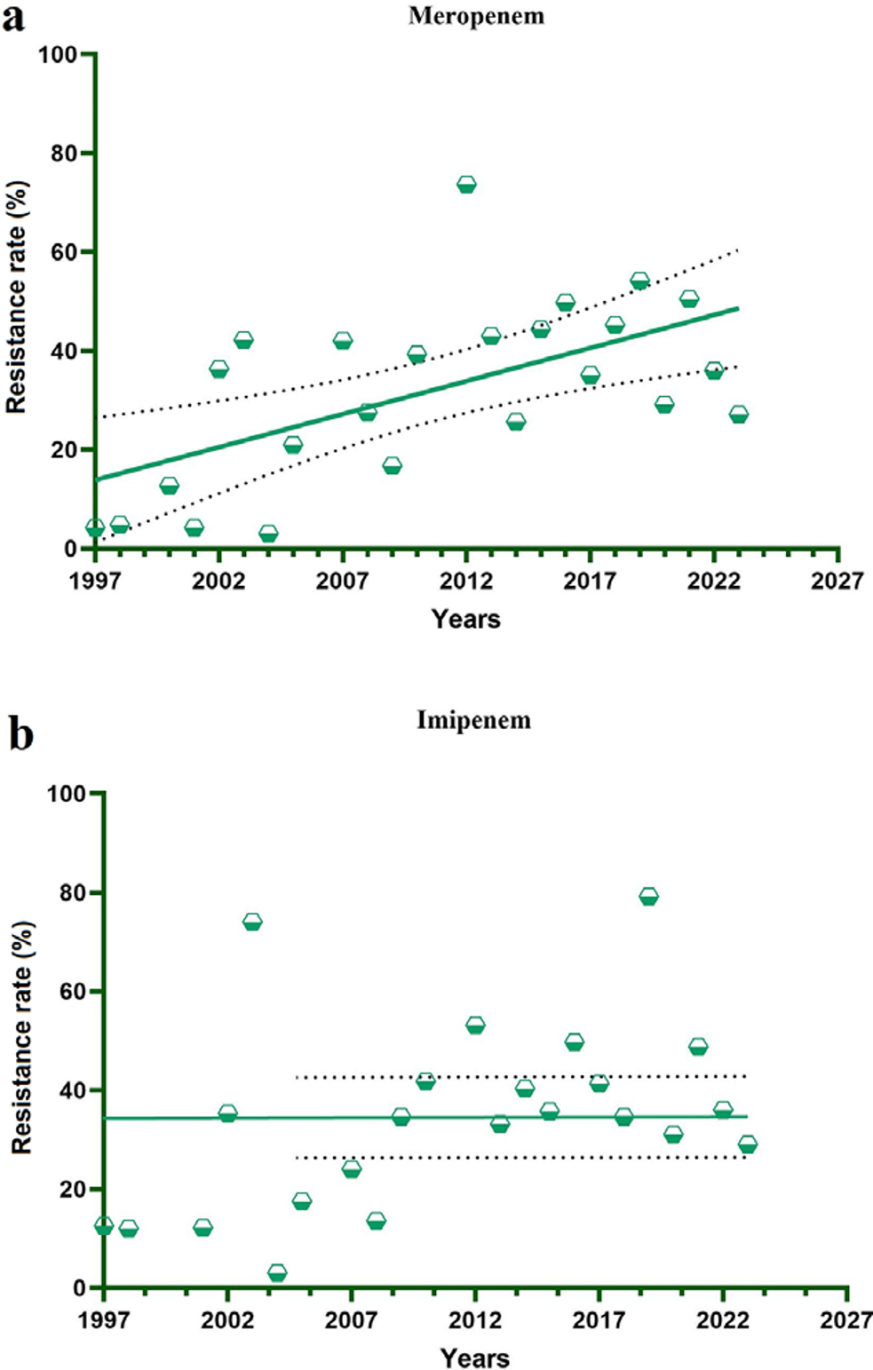
The resistance rates of *P. aeruginosa* to meropenem (a) and imipenem (b) during the years 1997 to 2023. Dashed lines represent 95% confidence intervals (CI: 0.2491–0.8040).

Time trend analysis (Pearson correlation) revealed a significant increase in *P. aeruginosa* resistance to meropenem from 1997 to 2023 (r = 0.593, p = 0.002, 95% CI: 0.2491–0.8040), indicating a strong correlation with year of publication. Figure 5 illustrates the resistance rates of imipenem and meropenem between 1997 and 2023.

**Figure 6.**
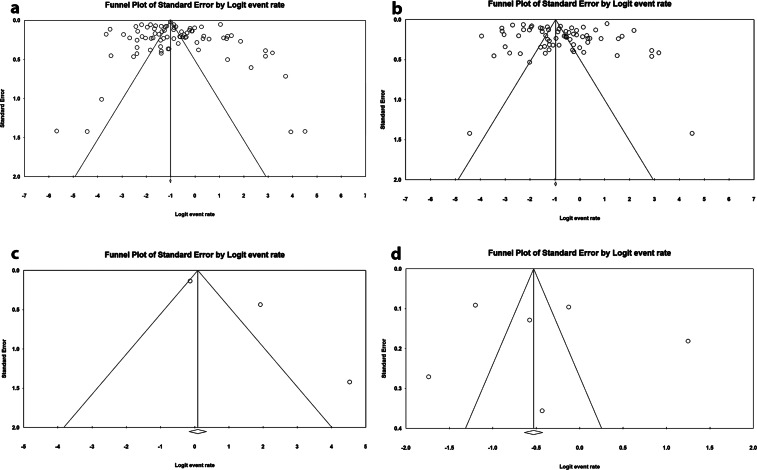
Funnel plots of the meta-analysis for the resistance rate of *P. aeruginosa* isolates to carbapenems: imipenem (a), meropenem (b), ertapenem (c), and doripenem (d).

Table 2 shows mid-range resistance rates in *P. aeruginosa* isolates: imipenem (50%), meropenem (50%), ertapenem (73.3%), and doripenem (46.3%). Significant heterogeneity was present across all studies (I^2^ > 50%, P < 0.05), but no publication bias was detected (Figure 6, Table 2, Egger’s and Begg’s tests). Fewer than 10 articles investigated ertapenem and doripenem resistance, precluding further analysis.

## Discussion

Our analysis found substantial geographic difference in *P. aeruginosa* resistance to clinically important carbapenems (imipenem, meropenem, ertapenem, and doripenem), with reliably elevated resistance rates observed globally. Carbapenem-resistant *P. aeruginosa* (CRPA) has been downgraded in WHO Bacterial Priority Pathogens List (BPPL) from ‘critical’ to ‘high priority’ in the 2024 update, reflecting a potential global decrease in resistance observed in at least one WHO region and its relatively lower transmission compared to other carbapenem-resistant strains. These results are consistent with resistance patterns reported in the Global Burden of Disease Antimicrobial Resistance study [105].

Ertapenem was investigated due to its potential to reduce the use of imipenem, meropenem, and doripenem in antimicrobial stewardship programs without increasing resistance in Enterobacteriaceae and *P. aeruginosa.* However, despite ertapenem’s potential benefit in mitigating carbapenem resistance in *P. aeruginosa*, our study revealed a scarcity of research evaluating its antimicrobial activity. This may be due to some authors supporting ertapenem as an approach to decrease resistance to meropenem and imipenem [106].

Based on our evaluations, contributor characteristics, situations, sample types, antimicrobial susceptibility procedures, sample sizes, and study preference were highly heterogeneous. Geographical differences in *P. aeruginosa* antibiotic resistance frequencies likely come from differing antibiotic prescribing and consumption patterns across populations. Therefore, the important CRPA burden in high-income areas, particularly Central/Eastern Europe and Central Asia, demands urgent continued investment in targeted antimicrobial development [105, 107, 108]. Furthermore, its high mortality rate among immunocompromised individuals and in healthcare settings necessitates ongoing innovative research and development to mitigate its impact. The WHO BPPL guides global surveillance by prioritizing critical threats [109, 110].

ß-Lactams are broad-spectrum antibiotics often used empirically for sepsis, with carbapenems possessing a particularly broad antimicrobial spectrum [111]. Carbapenem use in patients with extended-spectrum ß-lactamase-producing pathogen infections is linked to lower mortality, according to studies. Carbapenems are the preferred first-line antibiotics for sepsis, but their increasing global use poses a serious public health threat [111]. This threat requires that the WHO Global Antimicrobial Resistance and Use Surveillance System (GLASS) should continue monitoring CRPA for emerging resistance and intervention effectiveness [109].

The substantial heterogeneity and varying sample sizes across included studies may have influenced our analysis. The relative weight was calculated for each study and reported in the present study to overcome this problem. This study was limited by its restriction to English-language sources, potentially excluding relevant research. Furthermore, the meta-analysis, constrained by fewer than ten articles on *P. aeruginosa* antimicrobial resistance to ertapenem and doripenem, lacked statistical power and precluded definitive conclusions.

This meta-analysis was limited by the variation in breakpoints and methods used to define antimicrobial susceptibility and resistance across different studies. CLSI recommended standard-specific zone diameter and MIC breakpoints for imipenem and meropenem in *P. aeruginosa* antibiotic susceptibility testing in 2011 (M100-S21) and 2022 (M100-Ed32) [112, 113]. The meta-analysis included studies from 1997 to 2023. Studies prior to 2011 and 2022 used different antimicrobial susceptibility testing guidelines (non-M100-S21, M100-Ed32), resulting in variations in breakpoints and thresholds for susceptible and resistant strain determination.

The studies in our analysis exclusively used phenotypic methods for antibiotic susceptibility evaluation. These methods encompass classical culture-dependent techniques (disk diffusion assay, gradient diffusion method) yielding results in 18–48 h and automated microdilution systems providing faster results (6–24 h post-isolation). Fast bacterial identification and antibiotic susceptibility testing are crucial to combatting drug resistance, but traditional culture-based methods are time-consuming. Necessity often compels physicians to prescribe antibiotics empirically, contributing to inappropriate use, increased mortality and costs, and the spread of antibiotic resistance [114]. To expedite patient treatment and outcomes, continuous development of rapid antibiotic susceptibility and microorganism identification methods is crucial. Traditional methods like broth microdilution and disk diffusion remain essential in clinical practice to ensure accurate results that align with EUCAST and CLSI standards or to validate new methods [115].

Combating antibiotic resistance in *P. aeruginosa* and other pathogens necessitates coordinated global efforts, continuous monitoring, and responsive control programs. Developing new anti-Pseudomonas therapeutics is a key challenge in pharmaceutical science, alongside the pursuit of rapid diagnostic methods. Intensively studied methods for supporting the human immune system include preventative vaccines, therapeutic antibodies, and the development of antimicrobial peptides [116]. Antimicrobial agents encompass novel antibiotics, ß-lactamase and efflux pump inhibitors, quorum-dissolving molecules, and antibacterial nanoparticles. Developing alternative therapies like phage and photodynamic therapies, which employ mechanisms distinct from traditional antibiotics, is crucial.

In conclusion, the findings of the present study indicate that the overall resistance to carbapenems in clinical isolates of *P. aeruginosa* is relatively high and prevalent throughout the world. Moreover, time trend analysis showed that the resistance to meropenem has increased from the year 1997 to 2023. The increasing resistance of *P. aeruginosa* to carbapenems, as the last-resort antibiotics, is considered a global threat. Therefore, it requires new treatment approaches to fight against antimicrobial resistance and reduce the treatment failure. Moreover, applying the national and international surveillance programmes is necessary to control the emergence and spreading of carbapenem-resistant strains.

## Data Availability

The datasets supporting this study are included in the article, and excess information is available from the corresponding author upon request.
